# Navigating the complex currents of risk environments among people who use drugs: A commentary

**DOI:** 10.1177/14550725251396863

**Published:** 2025-11-13

**Authors:** Ola Røed Bilgrei, Kristin Hanoa

**Affiliations:** 1 Consumption Research Norway (SIFO), 60499 Oslo Metropolitan University, Oslo, Norway; 2 Department of Alcohol, Tobacco and Drugs, 25563 Norwegian Institute of Public Health, Oslo, Norway

In their article, “*A Strait or an Ocean? Exploring Risks and Resources among People who use Drugs in Denmark and Sweden*”, Houborg et al. (2025) explore how local drug-policy contexts influence risk environments among structurally vulnerable people who use drugs in Malmö and Copenhagen. Despite similar political backgrounds characterized by relatively comprehensive welfare states, the two cities exhibit notably different drug policy regimes. On the one hand, Sweden's drug policy remains defined by strict drug control and a limited focus on harm reduction. On the other hand, Denmark has traditionally pursued a more liberal approach to drug control and has been a pioneer in implementing harm reduction measures within the Nordic region. Against this backdrop, Houborg et al. (2025) offer a valuable contribution by exploring how these differing regimes shape the local risk environments experienced by people who use drugs.

Based on identical questionnaires used in structured interviews with people who use drugs in the two cities, the data are analysed quantitatively using descriptive statistics, and the results are presented comparatively across the variables “sociodemographics”, “drug use”, “marginalization and entrenchment”, “use of formal and informal resources” and “everyday concerns”. The authors found that participants from Copenhagen made greater use of available harm reduction services, while those from Malmö used drugs in riskier settings, relied more on peers for resources, and also expressed greater concern about overdoses, other drug-related harms and police arrest. After noting several methodological limitations, the authors cautiously conclude that there is “*a possible interplay between drug policy and the Nordic welfare state in shaping the risk environments and everyday lives of structurally vulnerable people who use drugs in Copenhagen and Malmö*” (Houborg et al., 2025, pp. 11–12). This conclusion is neither controversial nor groundbreaking. Nonetheless, as an initial attempt to understand the consequences of different drug-policy regimes, the article makes a valuable contribution by illuminating the complex interplay between Nordic welfare states, harm reduction measures and the local risk environments of people who use drugs.

To further situate the article's findings within a broader empirical context, Norway offers a particularly relevant case – positioned between the two poles of the Nordic drug policy spectrum exemplified by Sweden and Denmark. Similar to Denmark, Norway maintains an extensive social welfare system that provides a range of free services for people who use drugs, including drug treatment, opioid substitution treatment, needle and syringe exchange programs, drug consumption rooms, widespread distribution of intranasal naloxone, and, more recently, a time-limited trial project with heroin-assisted treatment (Madah-Amiri et al., 2017; Myklebust et al., 2024). Historically, however, Norwegian drug policy has been described as “schizophrenic” (Skretting, 2014), reflecting its simultaneous emphasis on criminal prosecution – akin to the more punitive approach long associated with Sweden – and on health-oriented interventions. Although this duality has become less pronounced in recent years, with a gradual shift from punitive measures toward more supportive approaches (Larsson, 2021), the risk environments of Norway's street-based drug scenes continue to produce additional harms (Hanoa et al., 2024).

Against this backdrop, a series of qualitative studies involving people who inject drugs in Norway (Bilgrei et al., 2024; Hanoa et al., 2024; Hanoa et al., 2022; Hanoa et al., 2023) explored a paradox that emerges from this policy landscape: despite the implementation of numerous harm reduction and preventive measures, Norway continues to report one of the highest and most stable overdose-related mortality rates in Europe. Importantly, these studies demonstrate that perceptions of drug-related risk are relational and socially contingent – learned, negotiated and reproduced through interactions within street-based drug scenes. Through these processes, people who use drugs construct notions of *acceptable risk*, allowing certain practices to be perceived as desirable, or even pleasurable. At the same time, contextual factors within their risk environments – such as distress, fear, and stigma – can foster more harmful patterns of use. The Norwegian case illustrates that risk environments are shaped not merely by the availability of welfare and health services, but by how individuals engage with, resist, or adapt to them within specific social and cultural contexts. These studies underscore that welfare-oriented policies are insufficient unless they meaningfully engage with the lived experiences, competencies and relational dynamics that structure everyday harm reduction practices. Consequently, prevention and harm reduction measures that focus narrowly on individual behavior change often prove ineffective, as people remain embedded in social contexts that constrain their capacity to act on such knowledge.

These insights help frame two key considerations relevant to the study by Houborg et al. (2025). First, policy matters. As Houborg et al. (2025) demonstrate, there are associations between drug-policies and practices of drug use: participants in Malmö used drugs in riskier settings and had less access to harm reduction services compared to their Copenhagen counterparts. Although methodological limitations prevent causal claims, the findings support the idea that drug policies interact with local risk environments among people who use drugs. Second, the Norwegian studies discussed above suggest a non-linear relationship between the availability of harm reduction or preventive measures and drug-related harms. This underscores the importance of attending to context – to further understand how drug-use behaviours are shaped, transformed and embedded within shared social and symbolic meanings. Consequently, street-level drug use should be analysed alongside the practices, interactions and behaviours of the individuals and groups who inhabit these environments (Duff, 2010), as well as the social circumstances that lead some users to “risking risk” (Lovell, 2002). This calls for greater qualitative sensitivity in understanding the risk environments among people who use drugs.

Therefore, Houborg et al. (2025) rightly argue that “*more comparative research is needed to explore how drug policy shape risk environments*” (p. 2). To navigate the complex currents of risk environments among people who use drugs, their study makes a valuable contribution in setting the benchmark for further research. Building on this foundation, future research should place greater emphasis on how structural conditions – particularly the availability and accessibility of harm reduction measures, alongside housing instability, policing practices and social stigma – intersect with individual and collective understandings of risk. Such an approach would deepen our understanding of how drug policy, social context and lived experience co-produce the harms and possibilities that define risk environments within the Nordic welfare states.
